# Evaluating the performance of Bayesian and frequentist approaches for longitudinal modeling: application to Alzheimer’s disease

**DOI:** 10.1038/s41598-022-18129-4

**Published:** 2022-08-24

**Authors:** Agnès Pérez-Millan, José Contador, Raúl Tudela, Aida Niñerola-Baizán, Xavier Setoain, Albert Lladó, Raquel Sánchez-Valle, Roser Sala-Llonch

**Affiliations:** 1 Alzheimer’s Disease and Other Cognitive Disorders Unit, Neurology Service, Hospital Clínic de Barcelona, Institut d’Investigacions Biomèdiques August Pi i Sunyer (IDIBAPS), Fundació Clínic per a la Recerca Biomèdica, Universitat de Barcelona, 08036 Barcelona, Spain; 2 Institute of Neurosciences. Department of Biomedicine, Institut d’Investigacions Biomèdiques August Pi i Sunyer (IDIBAPS), Faculty of Medicine, University of Barcelona, 08036 Barcelona, Spain; 3 Centro de Investigación Biomédica en Red de Bioingeniería, Biomateriales y Nanomedicina (CIBER-BBN), Barcelona, Spain; 4 Nuclear Medicine Department, Hospital Clínic Barcelona, Barcelona, Spain; 5 Centro de Investigación Biomédica en Red de Enfermedades Neurodegenerativas, CIBERNED, Madrid, Spain

**Keywords:** Medical research, Neurology, Mathematics and computing

## Abstract

Linear mixed effects (LME) modelling under both frequentist and Bayesian frameworks can be used to study longitudinal trajectories. We studied the performance of both frameworks on different dataset configurations using hippocampal volumes from longitudinal MRI data across groups—healthy controls (HC), mild cognitive impairment (MCI) and Alzheimer’s disease (AD) patients, including subjects that converted from MCI to AD. We started from a big database of 1250 subjects from the Alzheimer’s disease neuroimaging initiative (ADNI), and we created different reduced datasets simulating real-life situations using a random-removal permutation-based approach. The number of subjects needed to differentiate groups and to detect conversion to AD was 147 and 115 respectively. The Bayesian approach allowed estimating the LME model even with very sparse databases, with high number of missing points, which was not possible with the frequentist approach. Our results indicate that the frequentist approach is computationally simpler, but it fails in modelling data with high number of missing values.

## Introduction

The availability of longitudinal data-repeated measures of the same subjects over time-provides the opportunity to study trajectories of disease biomarkers. This offers an unquestionable value, as measures of change and evolution can complement cross-sectional analyses—mainly based on group differences at a specific time point—into the understanding of neurological diseases and the evaluation of disease-modifying treatments. However, real-life longitudinal databases are often characterized by high levels of noise, high variability, and missing points that lead to unbalanced data. All these factors represent a challenge when creating the models and often limit their interpretability. In this context, the use of linear mixed effects (LME) models offers a powerful and versatile framework for analysing longitudinal data, being more adequate than classical approaches such as repeated measures analysis of variance (ANOVA) or cross-sectional analysis of percent changes^1–3^.

In addition, when these biomarkers are obtained from neuroimaging data, there are additional challenges, as there are strong dependencies within subjects and timepoints. In this sense, besides the clear dependencies between the different measures of one subject, there are also dependencies between subjects that need to be modelled. LME models attempt to reconcile these schemes by combining fixed and random effects, where fixed effects are assumed to represent those parameters that are the same for the whole population, while random effects are group dependent variables assumed to consider the variance in the data explained over time and subject. In our case, the random effects will take into account the variability of the non-independent measures from different subjects^4–6^⁠.

After data modelling, LME models are usually followed by statistical inference procedures, which allow the researcher to generate questions about the model and to further evaluate their statistical significance and clinical relevance. In this sense, while statistical significance is well established to p-values < 0.05, or equivalent, the assessment of clinical relevance has not yet a standard analysis^7^. It has been suggested that clinical relevance can never be determined form p-values alone^8^, and complementary statistics should emerge to overcome this limitation in interpretability.

Historically, the dominant approach for performing the full procedure of LME modelling + statistical inference has been the Frequentist LME (FLME) approach. However, different methods using a Bayesian LME (BLME) approach have been suggested^3,9^. As suggested in the editorial of Anna G.M. Temp et al.^10^, Bayesian statistics can be used jointly with frequentist approaches to draw clinically relevant conclusions that can complement classical studies based uniquely on statistical significance.

In general words, the FLME approach is based on sampling distributions and on the Central Limit Theorem^11,12^, and it treats the population parameters of interest as fixed values^11^. While in BLME, parameters are estimated from the population distribution, given the evidence provided by the observed data^11^. BLME is considered a more natural approach to answer a question, since it estimates the parameters of interest directly from the population distribution instead of estimating them from the sampling distribution^13^. The Bayesian approach treats the parameters of interest as random variables that can be described with probability distributions^11^⁠. These posterior distributions can be compared directly without referring to statistical results of multiple tests. Overall, the differences in comparing frequentist vs Bayesian approaches in different fields have opened a debate in several fields^14–17^.

Alzheimer’s disease (AD) is clearly one of the research fields that will benefit from the development of longitudinal statistical methods. It is believed that AD is a slowly evolving process that likely begins years before the clinical symptoms are manifested^18,19^⁠. Therefore, there is a strong interest in identifying subjects at high risk before the full clinical criteria for AD dementia are met^20,21^⁠, as well as in giving reliable prognosis at the subject’s level. The existence of public available databases, such as the Alzheimer’s disease neuroimaging initiative (ADNI) has facilitated the definition and validation of neuroimaging biomarkers for AD^22^⁠. In this sense, the hippocampal volume (HV), derived from structural Magnetic Resonance Imaging (MRI) data, has become one of the most widely used biomarkers. Compared with healthy aging, HV is progressively affected in AD, being already reduced in patients with Mild Cognitive Impairment (MCI) due to AD and more strongly affected in advanced AD stages^23–25^.

In the recent years, the longitudinal trajectories of some AD biomarkers using frequentist approaches have been widely described^1,9,26^. On the other hand, the attempts to incorporate Bayesian statistics have shown promising results^2,3,9^. Even if frequentist and Bayesian schools represent two different schools of thinking, they often complement to each other. In the present work we analysed longitudinal MRI data from the ADNI dataset, using both FLME and BLME approaches. We performed simulations of real-life datasets derived from a public big database to explore the robustness of the methods with limited sample sizes and missing data using both approaches. Our goal was to evaluate the pros and cons of these approaches in real-life scenarios. For this, we create (simulate) datasets that incorporate the common handicaps found in clinical studies, e.g., low number of participants, missing data points or unbalanced sets with the aim to provide recommendations for further studies as regards the use of frequentist and Bayesian approaches, whilst illustrating the limitations of both approaches and bringing attention to statistical significance and clinical relevance.

## Materials and methods

### Data

We used longitudinal brain MRI data (T1-weighted scans, combining 1.5 and 3.0 Tesla) from the ADNI database (adni.loni.usc.edu). The ADNI was launched in 2003 as a public–private partnership, led by Principal Investigator Michael W. Weiner, MD. Including participants from ADNI-1, ADNI-GO, ADNI-2 and ADNI-3. Scans had been previously preprocessed with the FreeSurfer Longitudinal stream^27^⁠, as explained elsewhere^28^. We focus our analyses on the HV, as it is a common AD biomarker and we include the total intracranial volume (ICV), as a known confound in neuroimaging studies. Therefore, we downloaded HV, ICV and demographics from the data server.

We included AD dementia and MCI patients, as well as Healthy control (HC) participants, as labelled by the ADNI consortium^21^. According to their clinical evolution, we further created the following groups:

1. *Stable HC (sHC)* subjects who were diagnosed as HC throughout the follow-up period.
2. *Converter HC (cHC)* subjects who were diagnosed as HC at baseline and progressed to MCI or AD dementia.
3. *Stable MCI (sMCI)* subjects who were diagnosed as MCI throughout the follow-up period.
4. *Converter MCI (cMCI)* subjects who were diagnosed as MCI at baseline and progressed to AD dementia.
5. *AD* subjects who were diagnosed as AD at baseline.

We initially selected subjects having at least two acquisitions and we created several datasets as starting points. Tables 1 and 2 provide descriptive statistics of our initially selected longitudinal samples.

**Table 1:** Characteristics of the longitudinal ADNI sample used.

Variable	sHC	cHC	sMCI	cMCI	AD	p-value
N	273	78	361	319	219	
Baseline age (years)	74.3 ± 5.7	76.2 ± 5.1	72.9 ± 7.4	72.4 ± 7.5	74.7 ± 7.9	0.19
Sex (M/F)	142/131	40/38	212/149	184/135	123/96	0.41
APOE-e4 (nc/c)	207/66	52/26	203/158	121/198	64/155	< 0.0005

Baseline age values are in mean ± standard deviation. M = male, F = female, nc = non-carriers, c = carriers.

p-values indicate differences between group. We used ANOVA for baseline age, and Fisher’s exact test for the other data.

**Table 2:** Number of scans per time point by clinical group and time between scans.

Time point	sHC (N)	cHC (N)	sMCI (N)	cMCI (N)	AD (N)	Time from baseline (years)
Baseline	273	78	361	319	219	0.00
Year 0.5	243	71	326	274	187	0.51 ± 0.05
Year 1	234	70	294	275	173	1.01 ± 0.06
Year 2	206	61	233	240	97	2.02 ± 0.08

Time from baseline values are in mean ± standard deviation.

The datasets used for the different analyses were:

1. *Dataset 1* consisted of all the available data from the 4 timepoints, as described in Tables 1 and 2 (N = 1250 subjects).
2. *Dataset 2* was a reduced version of *dataset 1* containing only sMCI and cMCI subjects (N = 680 subjects).
3. *Dataset 3* was a homogeneous balanced database. We selected from *dataset 1*, subjects with 4 timepoints available. Demographics for this database are summarized in Table 3 (N = 670 subjects).
4. *Dataset 4* is a reduced version of *dataset 3* containing only sMCI and cMCI subjects (N = 373 subjects).

**Table 3:** Characteristics of the balanced longitudinal ADNI sample used.

Variable	sHC	cHC	sMCI	cMCI	AD	p-value
N	172	53	187	186	72	
Baseline age (years)	74.2 ± 5.6	76.1 ± 5.4	72.0 ± 6.9	71.6 ± 7.6	74.2 ± 7.9	0.004
Sex (M/F)	96/76	24/29	104/83	107/79	40/32	0.62
APOE-ɛ4 (nc/c)	133/39	33/20	116/71	72/114	21/51	< 0.0005

Baseline age values are in mean ± standard deviation. M = male, F = female, nc = non-carriers, c=carriers.

p-values indicate differences between group. We used ANOVA for baseline age, and Fisher’s exact test for the other data.

### Implementation of LME models

As there is not a fixed rule for choosing the number of random effects in LME, we evaluated two different models. Both models included the Intercept term, or group-mean, as a random effect. For the first LME model, the fixed effects were: time from baseline, group, group-by-time interaction, baseline age, sex, APOE status, APOE-by-time interaction and ICV. For the second LME model, the slope (measured as time from baseline) was also included as a random effect and the rest of variables were left as fixed effects (see Supplementary Material for details). The selection of the variables to be included in the models was done mimicking the analysis performed by Bernal-Rusiel et al. and according to previous AD literature^29,30^. HV (the outcome variable of our model) and ICV (a fixed effect variable of the model) variables were standardized to zero mean and standard deviation of one, using Fisher’s Z norm, to ensure that the estimated coefficients are all on the same scale and therefore the corresponding effect sizes are comparable.

### Statistical inference

We first studied which of the two proposed LME models were more appropriate for our sample using the frequentist approach with ANOVA and the Akaike Information Criteria (AIC). We used an ANOVA with χ^2^ test on the model parameters and coefficients estimated for both models and we assessed the significance with the likelihood ratio test^31^.

We then used frequentist statistical inference to test some of the well-known research questions in the AD field. For that, we created a set of contrasts using F-tests and using Satterthwaite’s method^32^ to compute the degrees of freedom. The contrasts studied were:

1. Are there differences across the 5 groups? (i.e., ANOVA main effect).
2. Are there differences between sMCI and cMCI?
3. Are there differences between cMCI and AD?
4. Are there differences between sHC and sMCI?
5. Are there differences between sHC and AD?
6. Are there differences between sHC and cMCI?
7. Are there differences between sHC and cHC?

We evaluated the LME model and tested these 7 contrasts in the datasets described previously (note that with dataset 2 or 4 we could only test contrast 1).

For the BLME approach, we also used the LME model with two random factors (the intercept and the slope). Posterior distribution measures regression parameters *ß* and contains all the information for statistical inference. We used the Credible Intervals (CrI) of this posterior distribution to study group differences. The CrI differ from the well-known Confidence Intervals (CI) in the fact that they are based in the uses of prior information and allow direct inferences about plausibility. Thus, CrI need the use of prior information to be estimated and can be interpreted as the probability in terms of plausibility^33^. We considered the four datasets and the same 7 contrasts described above.

All analyses were implemented in software R (https://www.r-project.org), version 3.6.2. For the LME model we used the *lme4* package^34^ and the *rstan* package^35^, so we combined R and Stan (https://mc-stan.org/) languages. The code for these analyses is available at https://github.com/Agnes2/LME-with-a-Bayesian-and-Frequentist-Approaches.git.

### Simulation of real-life databases

**Figure 1:**
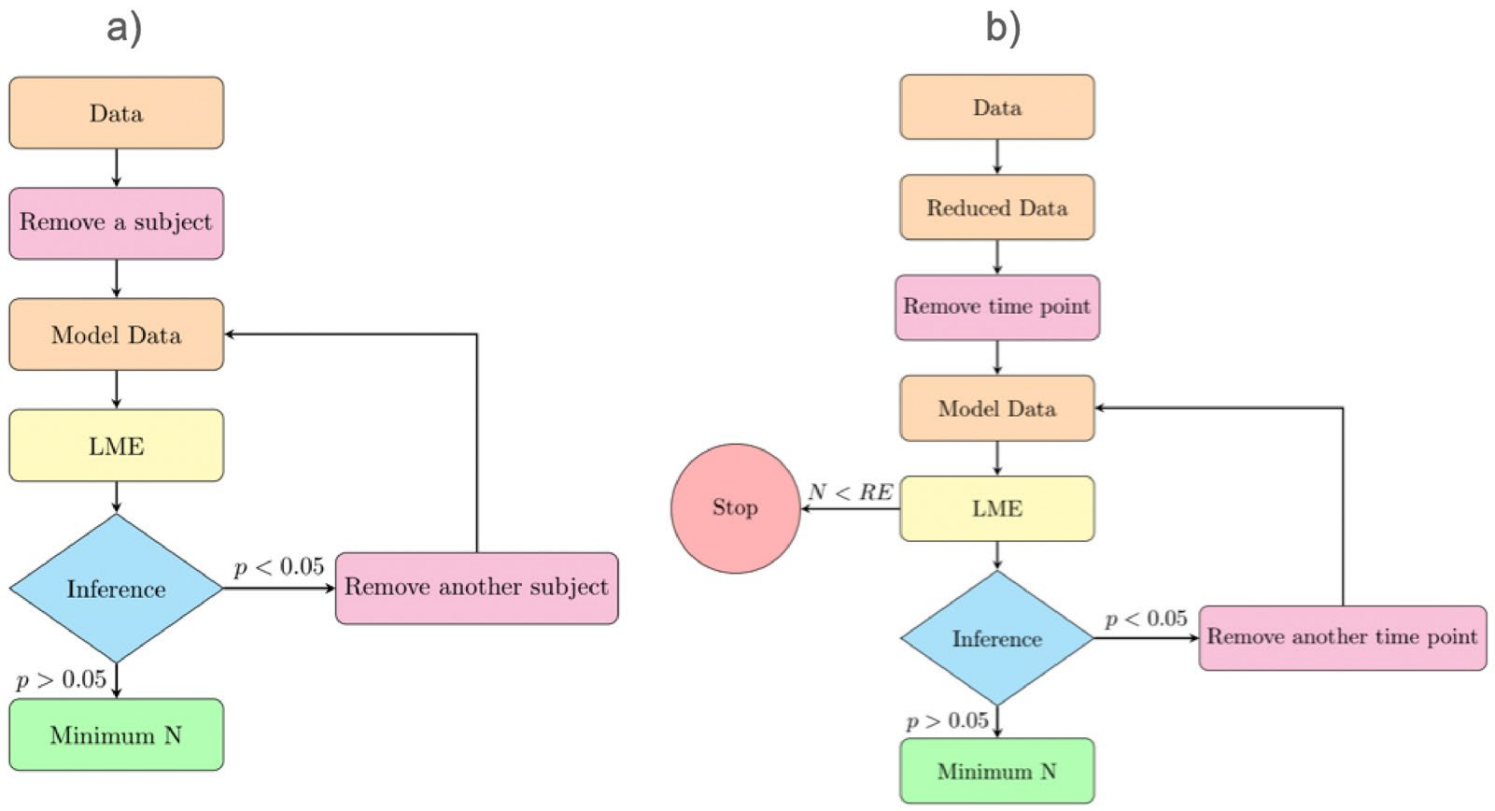
Simulations’ scheme (**a**) Strategy for minimum N simulations. The initial data is dataset 1 or dataset 2. (**b**) Strategy of the simulation of missing time points. *RE*= random effects.

Firstly, with the aim to provide a recommendation of the minimum N needed in these studies, we performed sequential simulations on the databases. We followed the scheme shown in Fig. 1a. We started from either dataset 1 (all groups) or dataset 2 (only MCI). We randomly selected one subject and we removed it (all its time points) from the dataset. Then we re-estimated the LME model, and we calculated the contrast of interest. This was repeated until the stopping criterion was met. At this point, we stored the last significant database, as a borderline significant dataset. Here, the stopping criterion was set at p-value > 0.05. This procedure was performed with dataset 1 (i.e., minimum N to differentiate across the 5 groups) and dataset *2* (i.e., minimum N to differentiate between sMCI and cMCI).

Further, with the aim to evaluate another typical situation in these studies, we also tested the effect of *missing timepoints*. We started from *dataset 3* (full balanced data) or *dataset 4* (MCI balanced data), and we proceeded as shown in Fig. 1b. First, we randomly selected one subject’s time point of the sample and we removed it. We then estimated the FLME model, and we performed the corresponding statistical inference. We progressively removed time points from different subjects until the stopping criterion was met, and, as above, the last database was kept as a borderline significant database. The stopping criterion was set at p-value > 0.05. However, it should be mentioned that the FLME model cannot handle having more subjects’ samples than random effects. Therefore, this restriction was added as an additional stopping criterion. The random effects were measured as *N* × *2* subjects’ samples, as we had a FLME model with randomly varying intercept and slope.

All the simulations were repeated over 500 iterations to account for the random selection of the subjects/timepoints to be removed at each step, leading to 500 *borderline significant databases*.

We applied BLME to the most compromised datasets found with FLME and we studied its behavior. Here, we performed a descriptive analysis in the borderline situations found with FLME. For that, we studied the obtained borderline datasets with the BLME model to estimate if they remained significant in the Bayesian framework and to evaluate the potential clinical interpretations that could be derived from them in terms of relevance.

## Results

### Statistics on ADNI longitudinal databases

Of the two possible LME models to fit our data—one with the intercept as a random effect and another with intercept and slope as random effects—we found that the second one performed better for explaining our data. This was verified by the results of the ANOVA (p-value <<< 0.001) and by comparing their AIC values (1546.2 vs 1411.7). Therefore, all further analyses were performed with this model. To obtain comparable results, we also used intercept and slope as random effects in the BLME model.

We applied the FLME model followed by a set of F-tests to evaluate the contrasts of interest in the four databases described above. Results are shown in Table 4, and they reproduce previous reports on the field (as those presented in Ref.^1^⁠). Mainly, we found significant differences in HV (*p*-value < 0.05) between all the five clinical groups, between sMCI and cMCI, between sHC and AD and between sHC and cMCI for the four initial datasets configurations. All these differences remained significant after correcting for multiple comparisons using Bonferroni (n = 7 tests, *p*-value < 0.05/7).

**Table 4:** Summary of the null hypotheses tested and results of the statistical inference.

Contrast	Dataset 1 F, p	Dataset 2 F, p	Dataset 3 F, p	Dataset 4 F, p
sMCI vs cMCI	39.2 5.6 × 10^–10^ *	36.1 3.2 × 10^–9^*	31.8 2.5 × 10^–8^*	24.2 1.3 × 10^–6^*
All groups	22.8 4.1 × 10^–18^ *	–	15.1 8.5 × 10^–12^*	–
AD vs cMCI	2.0 0.2	–	0.2 0.6	–
sHC vs sMCI	2.3 0.1	–	1.0 0.3	–
sHC vs AD	53.8 4.1 × 10^–13^*	–	27.7 1.9 × 10^–7^*	–
sHC vs cMCI	53.4 5.7 × 10^–13^*	–	40.4 3.8 × 10^–10^*	–
sHC vs cHC	2.3 0.1	–	2.7 0.1	–

*Indicates p-value < 0.05 (Bonferroni corrected).

After fitting the BLME model, we obtained the joint posterior probability of the parameters. Here, we were interested in the posterior probability distribution for the *ß*s, and we used the interval from 2.5th to 97.5th percentiles to obtain the 95% CrI^36^. We focused on the *ß*s that represented change over time for the different groups (with sHC being the reference group). Results for dataset 1 are shown in Table 5. We found that the effect of time was significant (i.e., the 95% CrIs did not contain zero) for cMCI and for AD, while it was not significant for cHC and sMCI. When comparing groups, which not contain the reference group, we considered that there were differences when the corresponding CrI did not overlap. The contrasts with significant differences are the same as those depicted by the FLME approach.

**Table 5:** Estimation and 95% Credible Intervals (CrI) of the ßs of interest LME model fitted with a Bayesian approach.

Parameter	Interpretation	Estimate	95% CrI	
ß_11_	cHC × time	− 0.03	− 0.06	0.01
ß_12_	sMCI × time	− 0.02	− 0.04	0.01
ß_13_	cMCI × time	− 0.08	− 0.11	− 0.06*
ß_14_	AD × time	− 0.11	− 0.13	− 0.08*

CrI borders are expressed as the 2.5% and 97.5% percentiles.

*Indicates that the effect is significant (i.e., CrI does not contain zero).

### Finding compromised datasets with FLME

#### Minimum N simulations

**Figure 2:**
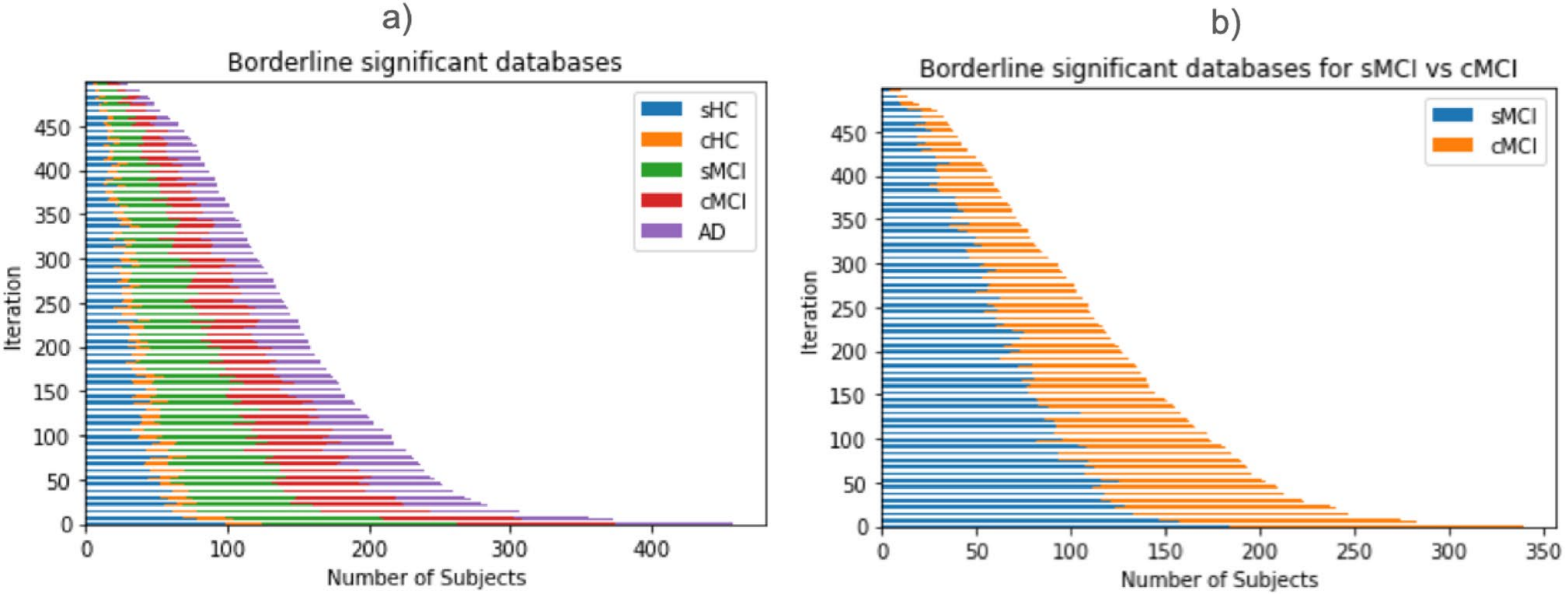
Distribution of subjects within each group for all the obtained databases (**a**) minimum N simulation across five clinical groups (**b**) minimum N simulation across MCI group.

By evaluating the 500 databases obtained from the procedure described in Fig. 1a and starting from dataset 1, we found that the minimum N needed to differentiate the five clinical groups (with p-value < 0.05) using the HV measure was N = 147 ± 73 overall. As the removal process followed a random order, the number of subjects within each group was not fixed by the algorithm. The group distribution resulting from the 500 databases is shown in Fig. 2a.

Similarly, with the same procedure and starting from database 2, we found that the minimum N needed to differentiate cMCI and sMCI using HV measures was N = 115 ± 64 overall. The distribution of the groups within the 500 obtained databases is shown in Fig. 2b.

#### Missing points simulations

For these simulations, in both cases (starting from *database 3* and *database 4*), we rapidly encountered that the limitation of number of samples < number of random effects. Thus, evidencing the low robustness of FLME approaches with highly unbalanced data.

By analysing the *database 3* (initial N = 670 with 4 time points per subject), with the process described in Fig. 1b, the simulations stopped at N = 612 ± 9 subjects (with different number of time points per subject), except from 3 iterations that did not converge into a failing database considered as outliers. At the moment that it was impossible to estimate the FLME model we had a mean of 2 missing time points per subject.

Similarly, with the same procedure and starting from *database 4* (initial N = 373 with 4 time points per subject) we found that the simulations stopped at N = 341 ± 7 except from 45 databases that did not stop. At the point that it was impossible to estimate the FLME model we had again a mean of 2 missing time point per subject.

### Evaluating compromised datasets with BLME

#### Minimum N simulations

**Figure 3:**
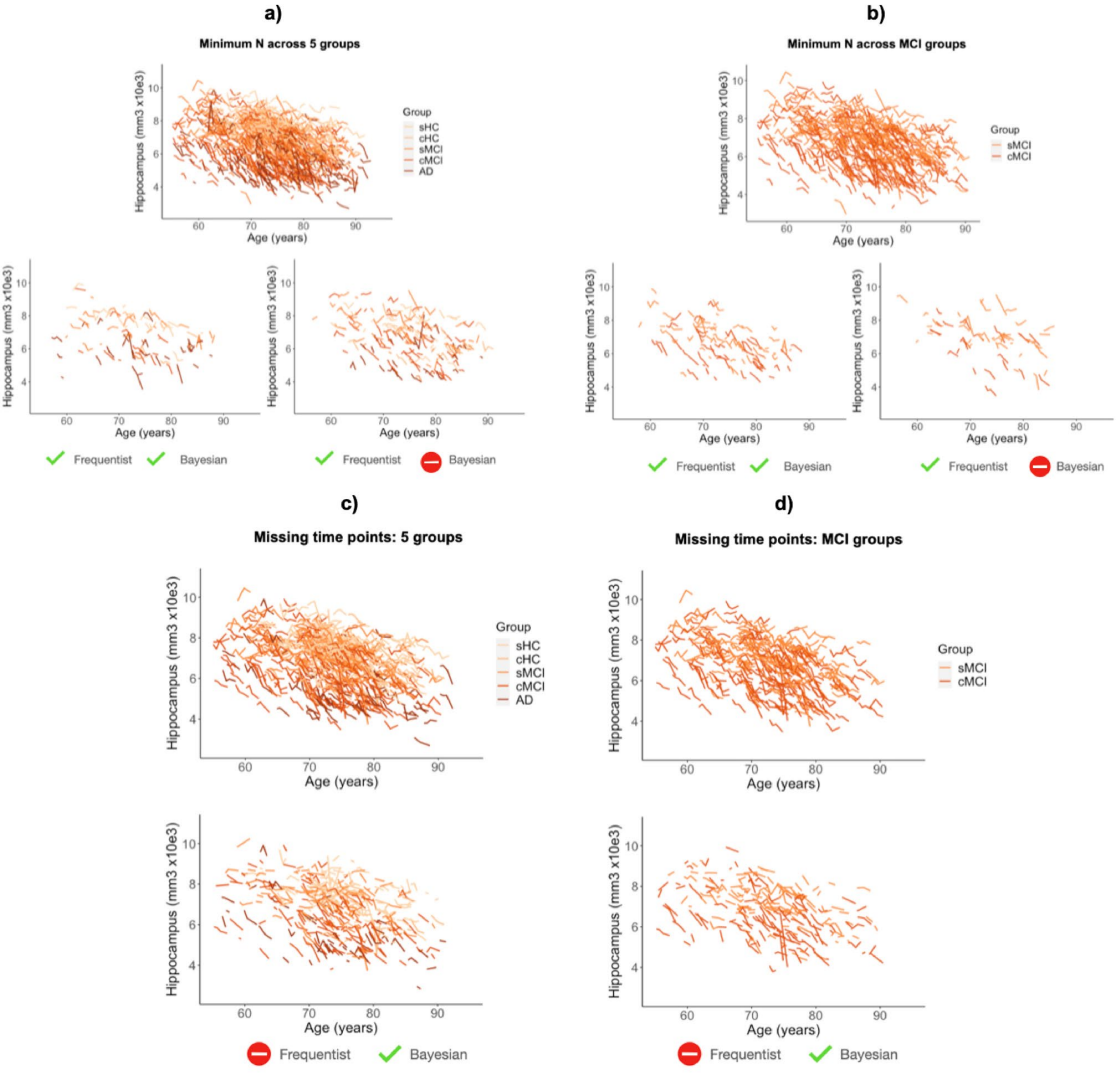
Hippocampus volume versus age. Top plots (**a,b**) represent dataset 1 (**a**) and 2 (**b**) with initial N. Bottom plots (**a,b**) represent four different databases obtained after the simulation of minimum N, resulting significant for frequentist and Bayesian approaches and only for frequentist approach. Top plots (**c,d**) represent dataset 3 (**c**) and 4 (**d**). Bottom plots (**c,d**) represent different databases obtained after the simulation of missing time points, being only estimable for Bayesian approach.

We studied the behaviour of BLME approach on different datasets obtained from the frequentist simulations of the minimum N. We first picked 10 different databases depicting differences across the 5 clinical groups, but that were at the limit for significance. These were randomly selected from the 500 iterations of the FLME experiments (the full characteristics of these databases are described in Supplementary Material). When studied with a BLME approach, 9 of them showed differences across the 5 groups. Figure 3a represents two of the datasets obtained in the simulation of the minimum N. Then, we selected 10 databases obtained from the simulations with dataset 2 (i.e., minimum N to find differences between sMCI and cMC). In this case, only 2 datasets remained significant when studied with the BLME approach. Figure 3b shows an example of the datasets obtained with the simulation of the minimum N.

#### Missing points simulations

To estimate the FLME model with a frequentist approach we encountered a practical limitation inherent to the model: the need to have more samples than random effects. Here, we selected 10 databases, from the FLME simulations, at the point that they no longer met the requirement. Thus, with these databases it was impossible to estimate a FLME model. The full characteristics of these databases are described in Supplementary Material. We studied with a BLME model these 10 databases. We found that the model can be estimated, and that all the databases depicted differences across the 5 groups (see Supplementary Material). For the 10 stopping databases created from the simulations with *dataset 4* we found similar results (see Supplementary Material), we could estimate the model and present differences for sMCI vs cMCI. Figure 3c,d represents Dataset 3 and 4 with one example of the datasets obtained after the missing points simulations for each situation.

## Discussion

In this study, we explored large longitudinal neuroimage datasets obtained from ADNI to study trajectories of hippocampal volume change in AD. For that, we created LME models under the frequentist and the Bayesian frameworks. We found that both approaches have similar behavior in finding differences with the entire database. In the minimum N simulations, the Bayesian approach was slightly stricter to significance when reducing data size. In addition, our results indicated that the Bayesian approach is more robust to unbalanced and sparse databases with different number of measurements across subjects.

Firstly, our investigation supports the use of LME approaches to model longitudinal data. The results of our null hypotheses testing agreed with those reported previously in AD for the hippocampus^1,2^. Additionally, we provide evidence of the utility of these apparently more complex analyses to study compromised datasets with different time points for across subjects.

The frequentist approach allowed us to implement a method for testing the relationship between the sample characteristics (size and missing points) and the expected group differences. Even considering that the statistical threshold (here p < 0.05) may be rather arbitrary (see^37,38^), it is important to note that this was chosen as a controlled systematic approach to study the behavior of the databases when removing subjects, with the ultimate goal to evaluate the behavior of both approaches in different scenarios. To our knowledge, there are no previous studies addressing similar questions with neuroimaging data.

In a further step, we aimed to explore the utility of Bayesian statistics combined with LME modelling. It has been suggested that Bayesian approaches could complement the findings obtained with frequentist analyses, as they provide a more interpretable framework. Bayesian models are based on the direct estimation from the population distribution represented by the posterior distribution, instead of estimating from the hypothetical sampling distribution as it happens in the frequentist approach^13^. In this context, our BLME model can be interpreted in a probabilistic way and may offer a more direct interpretation in clinical settings than FLME^13,14^. Contrarily, the FLME approach does not accept probabilistic interpretation although many researchers use them to interpret their results^39^. As we observed when testing large databases, the two approximations of LME model often led to similar conclusions. Indeed, our results in terms of statistical significance support previous research on the role of HV as a biomarker for AD, being highly significant across groups and between converters and non-converters. However, when repeating all comparisons with BLME, we aimed to add clinical relevance to the above significance statement. This was more evidenced when studying compromised datasets. That is, by using posterior distributions, longitudinal analyses can be better adapted to real-life datasets with clinical relevance. Overall, we emphasize the need of knowing the characteristics of the sample to be able to infer the correct interpretation of the results. For example, it is known that with a non-informative prior, Bayesian approaches tend to mimic frequentist results from the numerical point of view^11^. Little by little more studies use Bayesian approaches in the context of neuroimaging and dementia. In a similar context, Cespedes et al. demonstrated that the use of BLME can be useful for estimating atrophy rates in The Australian Imaging, Biomarker & Lifestyle Flagship Study of Ageing cohort (AIBL) (https://aibl.csiro.au). Bayesian statistics were used in the ADNI database in a latent time joint mixed-effects model to provide a continuous alternative to clinical diagnosis^40^. And, in a more complex approach, the authors implemented a multi-task Bayesian learning algorithm on the ADNI database to model trajectories of biomarkers at the individual level^9^. Although these studies clearly differ from ours, they support the use of Bayesian approaches in clinical contexts. In addition, Bayesian statistics appear as a good framework to solve clinically relevant questions that cannot be addressed with frequentist approaches. For example, the absence of effects^10^.

We calculated the minimum sample size that led to significant group differences with FLME, and we obtained values (overall values for the studied groups) of 147 and 115 for all groups and for MCI conversion to dementia respectively. It should be mentioned that the main goal of this study was not sample size estimation and thus, these values are rather indicative, in the sense that they are restricted to the research questions and the measure used in this study. However, we believe that they can be of interest in the context of clinical trials. In a study with frontotemporal dementia, Staffaroni et al. calculated the estimated sample size using bootstrapping techniques for different cognitive and imaging measures and they obtained values from < 100 to > 500 depending on the measure or combination of measures chosen^41^.

By studying highly compromised datasets (those at the border classical frequentist significance at p-value < 0.05), we were able to compare the two approaches. Notably, not all the borderline databases identified with FLME (i.e., p-value nearly 0.05) remained significant with the BLME approach. This may be due to the fact that accurate modelling of the variances in the Bayesian framework led to more restrictive statistics. It should be mentioned that the ADNI database is quite heterogeneous as do not have the same time point for each subject and that our analyses did not control for some external covariates such as different centers and scanners that might add variability.

In addition, it should be noted that our strategy for comparing approaches was based on selecting the datasets with FLME followed by the evaluation of significance with BLME. This strategy allowed us to obtain important insights as regards significance and interpretability for longitudinal modelling. However, the above conclusions are restricted to this and should not be generalized to any dataset.

The other group of simulations that we implemented was related to databases with missing points. In this sense, an important drawback for FLME modelling is the need of having more subjects’ samples than random effects for the model to be estimated. In practice, this was the main reason for stopping in these simulations. Instead, the Bayesian approach allowed estimating the LME model even with high number of missing points in the database. More specifically, our results show that the BLME model is feasible in a 4-timepoint database that has approximately 2 missing values for each subject, suggesting that the Bayesian framework should be chosen for longitudinal modelling in sparse databases. Other studies have demonstrated that Bayesian statistics overcome some of the limitations of classical statistical inference in non-homogeneous databases^3^.

Our study has several limitations. First, one difficulty of using the Bayesian approach is its complexity in computing posterior distributions used to estimate the CrI. This has historically imposed an important barrier^13^. Although software solutions have improved in the last years, the frequentist approach is still computationally easier. Further studies should explore the utilization of Markov Chain Monte Carlo approaches to overcome this limitation. Due to this high computational cost of the BLME, the implementation of a method for testing sequential data removal with BLME was out of reach of this study. Second, the current study is based on the HV measure, and the conclusions are specific for this. To be able to generalize our conclusions to broader contexts, other MRI biomarkers for AD, and eventually other databases, should be studied. Third, in the ADNI dataset, there are acquisition differences (i.e., different scanners) which were not included in the analyses. This could have an impact on the HV measurements. Finally, the clinical diagnosis available in the ADNI dataset does not systematically include CSF validation, which, according to the latest MCI diagnostic criteria^42,43^, may result in some subjects wrongly labelled as HC or MCI. Other sources of misclassification (or confusing diagnosis) refer to the fact that other pathologies may coexist in subjects diagnosed with AD and that different AD subtypes show different biomarker trajectories. Our results did not account for misdiagnosis nor subgrouping, as ground truth labels were not available. We believe that further studies, possibly using unsupervised machine learning, could account for these factors.

## Supplementary Information

Supplementary Information.

## Data Availability

Publicly available datasets were analyzed in this study. This data can be found at: adni.loni.usc.edu.

## Code availability

Analyses code is available at https://github.com/Agnes2/LME-with-a-Bayesian-and-Frequentist-Approaches.git.
