# Supplementary material for: Evaluating the performance of Bayesian and frequentist approaches for longitudinal modeling: application to Alzheimer’s disease
Source: Sci Rep. 2022 Aug 24;12:14448. doi: 10.1038/s41598-022-18129-4 (PMC9402558; doi:10.1038/s41598-022-18129-4)
Supplement: Supplementary file 1 — Supplementary Information. [file 41598_2022_18129_MOESM1_ESM.docx]

Supplementary Material

# Supplementary Figures and Tables

## Supplementary Tables

Supplementary Table 1: Number of subjects and scans per time point for databases studied with the BLME. The databases were obtained from the frequentist simulations of the minimum N for all the groups.

|  | ***N*** | ***sHC (N)*** | ***cHC (N)*** | ***sMCI (N)*** | ***cMCI (N)*** | ***AD (N)*** | ***Year 0 (N)*** | ***Year 0.5 (N)*** | ***Year 1 (N)*** | ***Year 2 (N)*** |
| --- | --- | --- | --- | --- | --- | --- | --- | --- | --- | --- |
| **Database 1** | 92 | 19 | 7 | 25 | 14 | 27 | 92 | 65 | 66 | 36 |
| **Database 2** | 189 | 31 | 12 | 55 | 51 | 40 | 189 | 154 | 128 | 96 |
| **Database 3** | 177 | 39 | 10 | 53 | 46 | 29 | 177 | 150 | 126 | 90 |
| **Database 4** | 161 | 35 | 8 | 43 | 40 | 35 | 161 | 128 | 115 | 87 |
| **Database 5** | 127 | 26 | 7 | 33 | 37 | 24 | 127 | 101 | 84 | 58 |
| **Database 6** | 182 | 41 | 9 | 51 | 45 | 36 | 182 | 146 | 130 | 89 |
| **Database 7** | 175 | 34 | 10 | 53 | 37 | 41 | 175 | 138 | 120 | 88 |
| **Database 8** | 97 | 22 | 6 | 24 | 23 | 22 | 97 | 73 | 71 | 44 |
| **Database 9** | 91 | 15 | 5 | 28 | 22 | 21 | 91 | 73 | 55 | 43 |
| **Database 10** | 156 | 29 | 5 | 47 | 43 | 32 | 156 | 127 | 110 | 64 |

Supplementary Table 2: Number of subjects and scans per time point for databases studied with the BLME. The databases were obtained from the frequentist simulations of the minimum N for MCI group.

|  | ***N*** | ***sMCI (N)*** | ***cMCI (N)*** | ***Year 0 (N)*** | ***Year 0.5 (N)*** | ***Year 1 (N)*** | ***Year 2 (N)*** |
| --- | --- | --- | --- | --- | --- | --- | --- |
| **Database 1** | 60 | 39 | 21 | 60 | 45 | 38 | 33 |
| **Database 2** | 123 | 62 | 61 | 123 | 102 | 92 | 65 |
| **Database 3** | 152 | 86 | 66 | 152 | 124 | 108 | 85 |
| **Database 4** | 128 | 68 | 60 | 128 | 107 | 92 | 77 |
| **Database 5** | 65 | 33 | 32 | 65 | 50 | 48 | 29 |
| **Database 6** | 96 | 53 | 43 | 96 | 81 | 70 | 47 |
| **Database 7** | 135 | 66 | 69 | 135 | 109 | 89 | 76 |
| **Database 8** | 74 | 38 | 36 | 74 | 54 | 43 | 38 |
| **Database 9** | 166 | 93 | 71 | 166 | 143 | 124 | 89 |
| **Database 10** | 133 | 78 | 55 | 133 | 107 | 92 | 71 |

Supplementary Table 3: Number of subjects and scans per time point for the stopping databases. 95% CrI of the ßs of interest (ß_11_: cHC x time, ß_12_: sMCI x time, ß_13_: cMCI x time and ß_14_: AD x time) LME model fitted with a Bayesian approach. CrI borders are expressed as the 2.5% and 97.5% percentiles. * Indicates that the effect is significant (i.e., CrI does not contain zero).

| ***Stopping Databases*** | ***sHC (N)*** | ***cHC (N)*** | ***sMCI (N)*** | ***cMCI (N)*** | ***AD (N)*** | ***Year 0 (N)*** | ***Year 0.5 (N)*** | ***Year 1 (N)*** | ***Year 2 (N)*** | ***95% CrI* ß_11_** | ***95% CrI* ß_12_** | ***95% CrI* ß_13_** | ***95% CrI* ß_14_** |
| --- | --- | --- | --- | --- | --- | --- | --- | --- | --- | --- | --- | --- | --- |
| **Database 1** | 158 | 45 | 173 | 178 | 67 | 309 | 298 | 314 | 321 | -0.10 0.03 | -0.04 0.04 | -0.09 -0.01 * | -0.15 -0.04 * |
| **Database 2** | 161 | 44 | 173 | 167 | 66 | 305 | 297 | 302 | 318 | -0.15 -0.03 * | -0.08 0.00 | -0.18 -0.10 * | -0.20 -0.08 * |
| **Database 3** | 152 | 42 | 167 | 166 | 64 | 304 | 307 | 286 | 285 | -0.10 0.04 | -0.07 0.03 | -0.13 -0.04 * | -0.13 0.01 |
| **Database 4** | 161 | 50 | 170 | 175 | 65 | 318 | 314 | 299 | 311 | -0.10 0.01 | -0.08 0.00 | -0.12 -0.04 * | -0.15 -0.04 * |
| **Database 5** | 166 | 46 | 169 | 170 | 67 | 338 | 302 | 295 | 301 | -0.11 0.02 | -0.06 0.02 | -0.14 -0.06 * | -0.14 -0.06 * |
| **Database 6** | 158 | 46 | 170 | 169 | 70 | 318 | 321 | 294 | 293 | -0.14 -0.02 * | -0.08 0.00 | -0.13 -0.04 * | -0.17 -0.06 * |
| **Database 7** | 165 | 47 | 169 | 173 | 65 | 323 | 302 | 299 | 314 | -0.09 0.03 | -0.05 0.03 | -0.12 -0.04 * | -0.15 -0.05 * |
| **Database 8** | 160 | 49 | 174 | 169 | 68 | 314 | 306 | 329 | 291 | -0.15 -0.02 * | -0.06 0.03 | -0.15 -0.06 * | -0.19 -0.06 * |
| **Database 9** | 157 | 47 | 174 | 172 | 67 | 323 | 303 | 306 | 302 | -0.11 0.02 | -0.04 0.04 | -0.12 -0.03 * | -0.12 -0.01 * |
| **Database 10** | 150 | 49 | 171 | 168 | 71 | 298 | 299 | 302 | 319 | -0.06 0.07 | -0.04 0.04 | -0.09 0.00 | -0.12 -0.01 * |

Supplementary Table 4: Number of MCI subjects and scans per time point for the stopping databases. 95% CrI of the ß of interest (cMCI x time) fitted with a Bayesian approach. CrI borders are expressed as the 2.5% and 97.5% percentiles. * Indicates that the effect is significant if the CrI does not contain zero.

| ***Stopping Databases*** | ***sMCI (N)*** | ***cMCI (N)*** | ***Year 0 (N)*** | ***Year 0.5 (N)*** | ***Year 1 (N)*** | ***Year 2 (N)*** | ***95% CrI*** |
| --- | --- | --- | --- | --- | --- | --- | --- |
| **Database 1** | 177 | 167 | 172 | 162 | 182 | 172 | -0.12 -0.03 * |
| **Database 2** | 169 | 175 | 174 | 163 | 173 | 178 | -0.13 -0.02 * |
| **Database 3** | 178 | 173 | 166 | 188 | 171 | 177 | -0.15 -0.05 * |
| **Database 4** | 172 | 172 | 181 | 164 | 171 | 172 | -0.14 -0.04 * |
| **Database 5** | 169 | 169 | 169 | 157 | 170 | 180 | -0.14 -0.05 * |
| **Database 6** | 176 | 172 | 170 | 156 | 188 | 182 | -0.13 -0.03 * |
| **Database 7** | 174 | 174 | 163 | 172 | 191 | 170 | -0.10 -0.01 * |
| **Database 8** | 179 | 173 | 161 | 194 | 185 | 164 | -0.10 -0.02 * |
| **Database 9** | 170 | 178 | 176 | 175 | 156 | 189 | -0.09 -0.01 * |
| **Database 10** | 172 | 168 | 157 | 167 | 179 | 177 | -0.14 -0.03 * |

# Supplementary Methods

Exact expression for the models of the LME models of the Section 2.2

The model with only intercept as random effect can be written as follows, where *i* indicates the subject and *j* indicates the time point:

$$Y_{ij}=\beta_{1}+\beta_{2}{cHC}_{i}+\beta_{3}{time}_{ij}+\beta_{4}{sMCI}_{i}+\beta_{5}{cMCI}_{i}+\beta_{6}{AD}_{i}+\beta_{7}APOE4_{i}+\beta_{8}{SEX}_{i}$$

$$+\beta_{9}{AGE}_{i}+\beta_{10}{ICV}_{i}+\beta_{11}{cHC}_{i}{time}_{ij}+\beta_{12}{sMCI}_{i}{time}_{ij}+\beta_{13}{cMCI}_{i}{time}_{ij}+\beta_{14}{AD}_{i}{time}_{ij}$$

$+\beta_{15}APOE4_{i}{time}_{ij}+b_{1i}+e_{ij}$

While the model with intercept and slope as random effects can be written as:

$$Y_{ij}=\beta_{1}+\beta_{2}{cHC}_{i}+\beta_{3}{time}_{ij}+\beta_{4}{sMCI}_{i}+\beta_{5}{cMCI}_{i}+\beta_{6}{AD}_{i}+\beta_{7}APOE4_{i}+\beta_{8}{SEX}_{i}$$

$$+\beta_{9}{AGE}_{i}+\beta_{10}{ICV}_{i}+\beta_{11}{cHC}_{i}{time}_{ij}+\beta_{12}{sMCI}_{i}{time}_{ij}+\beta_{13}{cMCI}_{i}{time}_{ij}+\beta_{14}{AD}_{i}{time}_{ij}$$

$+\beta_{15}APOE4_{i}{time}_{ij}+b_{1i}+b_{2i}{time}_{ij}+e_{ij}$

With the following variables:

*Y_ij_*: The variable to predicted. Here, HV.

*time_ij_*: time from baseline (in years).

*cHC_i_*=1: if subject is cHC and 0 otherwise.

*sMCI_i_*=1: if subject is sMCI and 0 otherwise.

*cMCI_i_*=1: if subject is cMCI and 0 otherwise.

*AD_i_*=1: if subject is AD and 0 otherwise.

*APOE4_i_*=1: if subject has at least one e4 allele and 0 otherwise.

*SEX_i_*=1: if subject female and 0 otherwise.

*AGE_i_*: age at baseline (in years).

*ICV_i_*: total intracranial volume (in litres).

*b_1i_*: random effect for the intercept term.

*b_2i_*: random effect for the slope term.

*e_ij_*: error term
